# Crosstalk Between LXR and Caveolin-1 Signaling Supports Cholesterol Efflux and Anti-Inflammatory Pathways in Macrophages

**DOI:** 10.3389/fendo.2021.635923

**Published:** 2021-05-27

**Authors:** Cristina M. Ramírez, Marta Torrecilla-Parra, Virginia Pardo-Marqués, Mario Fernández de-Frutos, Ana Pérez-García, Carlos Tabraue, Juan Vladimir de la Rosa, Patricia Martín-Rodriguez, Mercedes Díaz-Sarmiento, Uxue Nuñez, Marta C. Orizaola, Paqui G. Través, Marta Camps, Lisardo Boscá, Antonio Castrillo

**Affiliations:** ^1^ Instituto Madrileño de Estudios Avanzados (IMDEA) Research Institute of Food and Health Sciences, Madrid, Spain; ^2^ Unidad de Biomedicina (Unidad Asociada al CSIC), Instituto Universitario de Investigaciones Biomédicas y Sanitarias (IUIBS) de la Universidad de Las Palmas de Gran Canaria, Las Palmas, Spain; ^3^ Departamento de Morfología, Universidad de Las Palmas de Gran Canaria, Las Palmas, Spain; ^4^ Instituto de Investigaciones Biomédicas “Alberto Sols”, Consejo Superior de Investigaciones Científicas CSIC-Universidad Autónoma de Madrid, Madrid, Spain; ^5^ Institut de Biomedicina (IBUB), Universitat de Barcelona, Barcelona, Spain; ^6^ Centro de Investigación en Red sobre Enfermedades Cardiovasculares (CIBERCV), Madrid, Spain

**Keywords:** gene expression, caveolin-1, cholesterol efflux, inflammation, LXR, macrophage

## Abstract

Macrophages are immune cells that play crucial roles in host defense against pathogens by triggering their exceptional phagocytic and inflammatory functions. Macrophages that reside in healthy tissues also accomplish important tasks to preserve organ homeostasis, including lipid uptake/efflux or apoptotic-cell clearance. Both homeostatic and inflammatory functions of macrophages require the precise stability of lipid-rich microdomains located at the cell membrane for the initiation of downstream signaling cascades. Caveolin-1 (Cav-1) is the main protein responsible for the biogenesis of caveolae and plays an important role in vascular inflammation and atherosclerosis. The Liver X receptors (LXRs) are key transcription factors for cholesterol efflux and inflammatory gene responses in macrophages. Although the role of Cav-1 in cellular cholesterol homeostasis and vascular inflammation has been reported, the connection between LXR transcriptional activity and Cav-1 expression and function in macrophages has not been investigated. Here, using gain and loss of function approaches, we demonstrate that LXR-dependent transcriptional pathways modulate Cav-1 expression and compartmentation within the membrane during macrophage activation. As a result, Cav-1 participates in LXR-dependent cholesterol efflux and the control of inflammatory responses. Together, our data show modulation of the LXR-Cav-1 axis could be exploited to control exacerbated inflammation and cholesterol overload in the macrophage during the pathogenesis of lipid and immune disorders, such as atherosclerosis.

## Introduction

Liver X Receptors (LXRα and LXRβ) are transcription factors that belong to the nuclear receptor superfamily. These are endogenously activated by oxidized forms of cholesterol (oxysterols), and function as intracellular sensors of cholesterol levels (1). The accumulation of cholesterol in macrophages, derived from the uptake of lipoproteins or cellular debris, leads to LXR activation and triggers the induction of a transcriptional program to promote cholesterol utilization. One of these pathways promotes the export of cholesterol and phospholipids outside the cell, through the transcriptional induction of members of the ATP binding cassette family such as ABCA1 and ABCG1 (2, 3). Indeed, the capacity of LXR synthetic ligands to inhibit the development of atherosclerosis in mice results, in part, by promoting the exit of excess cholesterol from lipid loaded macrophages or foam cells, known as cholesterol efflux (4). In addition to their important role in cholesterol metabolism, LXRs are also involved in inflammation and in the regulation of immune responses. Activation of LXR with synthetic ligands has been shown to repress the expression of inflammatory genes in macrophages such as inducible nitric oxide synthase (iNOS), COX-2 or pro-inflammatory cytokines including interleukin-6 (IL-6) and interleukin-1β (IL-1β) induced by bacteria or lipopolysaccharide (LPS) (5, 6). Several mechanisms have been proposed to explain the anti-inflammatory actions of LXR ligands, including transrepression, or the induction of anti-inflammatory molecules. Importantly, Ito et al. elegantly described a mechanism underlying this antagonism, which appears to involve the LXR ligand-dependent induction of ABCA1 and the redistribution of membrane lipids thereby resulting in reduced inflammatory signaling (7).

Caveolin-1 protein (Cav-1) is the most common isoform responsible for the formation of caveolae, a type of invaginated lipid raft microdomains between 50-100 nm of the plasma membrane enriched in cholesterol, which play an important role in the regulation of various cellular functions including endocytosis, transcytosis and cellular signaling (8). Cav-1 is also a high affinity cholesterol-binding protein. In fact, the formation of caveolae and the expression of caveolin-1 are highly dependent on the availability of cholesterol (9, 10). Previous studies have shown that these proteins participate in the regulation of plasma lipoprotein metabolism, as well as cholesterol homeostasis, a process that must be adequately controlled to limit and avoid cholesterol accumulation and, ultimately, prevent the development of atherosclerosis (11). In this context, Cav-1 has been shown to participate in intracellular trafficking of *de novo* synthesized cholesterol to the plasma membrane (12–15). Therefore, it is believed that the deficiency of Cav-1 would lead to the accumulation of cholesterol in certain intracellular compartments (16, 17). Other studies have also suggested that Cav-1 can participate in cholesterol efflux to extracellular acceptors (11, 18), however, very little is known about the role of Cav-1 in cholesterol efflux induced by LXR-ABCA1/G1 in macrophages. Despite that the presence of caveolae-like invaginations in immune cells has been a controversial topic (19), and that the major expression of Cav-1 is primarily found in vascular endothelial cells, there is ample evidence demonstrating that Cav-1 is also expressed and functional in different immune cells, including macrophages (20–26). Nevertheless, although several studies in the last two decades reported different roles for Cav-1 in the context of macrophage biology, the role of Cav-1 in macrophages is not completely understood. In these cells, Cav-1 appears to participate in apoptosis, lipid and cholesterol metabolism, as well as an anti-inflammatory mediator (27–29). Importantly, Cav-1 expression has been shown to increase in response to LPS but the overall participation of this protein in immune processes is not entirely clear (30, 31). Since dysregulation of lipid and immune homeostasis in macrophages are contributing factors for the development of several chronic diseases, our study aims to explore the possible participation of Cav-1 in the metabolic and inflammatory effects of LXR in macrophages.

## Material and Methods

### Animal Procedures

Cav-1 deficient mice (Cav-1^−/−^, strain Cav-1tm1Mls/J, genetic background 129/Sv, C57BL/6J, and SJL) and their corresponding controls Cav‐1^+/+^ were obtained from the Jackson Laboratory (Bar Harbor, ME USA). LXRαβ^+/+^ and LXRαβ^-/-^ mice (Sv129/C57bl/6 background), were provided by David Mangelsdorf (University of Texas Southwestern, USA) and were maintained on standard chow under pathogen-free conditions. Mice aged 8-12 weeks were used for experimental procedures following Institutional Care Instructions (Bioethical Commission from Consejo Superior de Investigaciones Científicas).

### Antibodies

Antibodies against caveolin-1, flotillin-1, clathrin, total STAT3 and STAT1 and their phosphorylated forms were obtained from BD Transduction Laboratories (Lexinton, KY). Monoclonal anti β−Actin was purchased from Sigma-Aldrich (St. Louis, MO) and polyclonal anti-iNOS, anti-GFP and anti-COX2 were obtained from Santa Cruz Biotechnology. ABCG1 Antibody (NB400-132) was from Novus Biologicals. ABCA1 and F4/80 were detected using specific anti serums, kindly provided by Michael L. Fitzgerald and Mason W. Freeman (MGH, Boston MA), and Siamon Gordon (Oxford University), respectively. Alexa Fluor-conjugated secondary antibodies were from Molecular Probes (Eugene, OR). Horseradish peroxidase HRP- and gold-conjugated secondary antibodies were from Jackson Inmmunoresearch Laboratories (West Grove, PA).

### Cell Culture and Treatments

Macrophage cell line RAW 264.7 was obtained from the American Type Culture Collection. RAW 264.7 cells were cultured in Dulbecco’s modified Eagle’s medium (DMEM) containing 10% fetal bovine serum (FBS) 100 units/ml penicillin and streptomycin and 2mM L-glutamine at 37°C in a humidified incubator at 5% CO_2_ 95% O_2_. Culture reagents were purchased from BioWhittaker (Walkersville, MD). Mouse peritoneal macrophages were isolated from mice by peritoneal lavage using PBS 4 days after an intraperitoneal injection of 1.5 ml of sterile thioglycollate broth (thioglycollate-elicited). Macrophages were seeded in 35-mm dishes at 3x 106/well in RPMI 1640 medium supplemented with 10% FBS and penicillin/streptomycin (each at 100 units/ml). Unattached cells were washed off after 3 h with RPMI (twice), and the remaining macrophages were incubated overnight in RPMI supplemented with 10% FBS. On the following day, cells were washed twice again, incubated in 0.5% FBS RPMI, and treated as indicated. For cell treatments we used the specific LXR agonists GW3965, provided by Tim Willson and Jon Collins (GlaxoSmithKline). T0901317 and the RXR agonist, LG268 obtained from TOCRIS. Ligands were dissolved in DMSO before use in cell culture. LXR ligands were used at 1 µM, whereas RXR ligand was used at 50 nM. LPS from E. Coli was purchased from Sigma.

### Transfections

The full length murine Caveolin-1 or GFP cDNA were cloned in-frame in pCDNA3.1 vector (Invitrogen, Inc.). Twenty-four hours before transfection, 2x10**^6^** RAW 264.7 cells were plated per 60-mm dish. On the day of transfection, 5 µg of plasmid DNA was diluted in 200 µl of serum-free DMEM media. In a separate tube, 20 µl of Lipofectamine 2000 was diluted in 200 µl of serum-free DMEM media. The diluted DNA and Lipofectamine were then gently mixed and incubated at 25°C for 30 min. After the incubation, 6 ml of serum-free RPMI media was added to the DNA/Lipofectamine mixture, mixed, and placed onto cells rinsed with serum-free DMEM media. The cells were incubated for 5 h at 37°C. Without washing, 3.6 ml of DMEM media containing 20% FBS was added. The cells were grown for 24 h. The media was removed, and DMEM media containing 10% FBS and 1.5 mg/ml Geneticin (G418) was added. Different clones of antibiotic-resistant cells were isolated and tested for Cav-1 and GFP expression. Cells were grown under constant selection in medium containing 500 µg/ml Geneticin.

### Total Protein Extracts and Microsomal Purification

For total protein extracts, cells were washed in PBS and scraped with lysis buffer (62.5 mM Tris-HCl, 1% SDS, 60 mM octyl-glucoside 10% glycerol, pH 6.8) containing protease inhibitors (Roche, Boehringer Mannheim). To obtain liver protein extracts, the tissue was briefly dissected and washed thoroughly with ice cold phosphate buffered saline and snap frozen in liquid nitrogen. Frozen tissue was mechanically homogenized in lysis buffer with protease inhibitors. An aliquot of each extract was preserved for protein quantification by bicinchoninic acid assay (32). Five per cent β-mercaptoethanol and 0.001% bromophenol blue were then added, and samples were boiled at 95°C for 5 min. For microsomal fractionation, cells plated in three 100-mm wells were washed twice with ice-cold PBS and scraped down into PBS containing a protease inhibitor mixture. Cells were then sedimented and resuspended in 1 ml of hypotonic buffer (0.25 M sucrose, 20 mM Tricine, pH 7.8, 1 mM EDTA) with protease inhibitors. After 20 min on ice, cells were disrupted using a Potter-Elvehjem homogenizer. Nuclei and cellular debris were removed by sedimenting the homogenate at 1,000 g for 10 min at 4°C. The supernatant was ultracentrifuged 1h at 100,000 g in TLA-100.1 rotor (Beckman, Palo Alto, CA). The precipitated fraction was resuspended in lysis buffer and solubilized at 4°C.

### Detergent-Free Caveolae Extraction

Caveolae extraction from mouse peritoneal macrophages and Raw 264.7 cell cultures were carried out following the procedure described previously were TX-100 was replaced by sodium carbonate and a sonication step was introduced to finely disrupt cellular membranes (33). Briefly, cells were homogenized using a loose-fitting Dounce homogenizer (10 strokes) and a sonicator (three 20-s bursts). 5mg of cellular homogenate was then adjusted to 45% sucrose by the addition of 2 ml of 90% sucrose prepared in MBS (25 mM MES, pH 6.5, 150mM NaCl) and placed at the bottom of an ultracentrifuge tube. 5mg of cellular homogenate was transferred to a SW41-Ti tube. A 5-35% discontinuous sucrose gradient was formed above and centrifuged at 39,000 rpms for 20 h in an SW41 rotor (Beckman Instruments, Palo Alto, CA) to obtain a total of 13 fractions A light-scattering band confined to the 5–35% sucrose interface was observed that contained caveolin but excluded most other cellular proteins. A volume of 20 µl form each sucrose fraction obtained was analyzed by western blot with specific antibodies.

### Protein Analysis by Western Blotting and Bioplex ELISA

Equal amounts of each sample (25-50 µg) were electrophoresed on sodium dodecyl sulfate–polyacrylamide gels electrophoresis (SDS–PAGE) and transferred to nitrocellulose membranes. Membranes were pre-incubated with 5% blotting grade blocker non-fat dry milk (Bio-Rad Laboratories, Hercules, CA, USA) in TBS with 0.1% Tween 20 (TBS-T) at room temperature for 1 h and blotted over night at 4°C with the specific primary antibodies. Antibody-specific labeling was revealed by incubation with a HRP-conjugated goat anti-mouse or anti-rabbit secondary antibody (1:5000) and visualized with the ECL chemiluminescence kit (Amersham Biosciences). Cytokine production *in vitro* by macrophages and its accumulation in the culture medium was quantified using an ELISA Bioplex kit (Bio-Rad) according to the manufacturer’s instructions.

### RNA Isolation and Gene Expression

RNA from liver and peritoneal macrophages were extracted using Trizol reagent (Life Technologies, Inc). Levels proinflammatory genes like IL-6, IL-1β, iNOS, COX-2, and other mRNAs (caveolin-1 and ABCA1) were determined by quantitative reverse transcription real time (RT)-PCR (SYBRgreen) containing sense and antisense primer sequences of the tested genes as well as of 36B4 ribosomal protein (housekeeping gene) as we have previously described (34).

### Immunofluorescence Microscopy

Peritoneal macrophages and Raw 264.7 cells were seeded on coverslips at a density of 0.5 x 10^6^/well. For fluorescent labeling of lipid rafts, the cells were incubated with 2 µg/ml of CTxβ Alexa 594 (Molecular Probes, Inc.) in 0.1% BSA-PBS for 20 min at 4°C prior to cell fixation. Cells were then fixed in 4% paraformaldehyde (PFA) and permeabilized with 100% methanol for 5 min at -20°C. For liver immunohistochemical analysis, tissues were dissected from the animal, embedded in OCT compound (Tissue-Tek) and snap-frozen in liquid nitrogen and isopentane. 4 μm sections were air-dried, fixed with 4% PFA and permeabilized with 0.01% Triton X-100 in PBS. The sections and the coverslips were then sequentially incubated at room temperature in PBS containing 4% goat serum and 0.8% bovine serum albumin (BSA) for 60min, with the indicated primary antibody over night at 4°C and with the fluorescence-tagged secondary antibodies in 0.8% BSA-PBS for 60 min. The coverslips were then mounted on glass slides using Vectashield mountain medium fluorescence with DAPI (Vector Laboratories) and fluorescence signals were monitored using a Zeiss LSM 5 PASCAL Laser Scanning Microscope (Carl Zeiss, Germany).

### Electron-Microscopy Procedures

Culture cells were chemically fixed at 4°C with a mixture of 2% PFA and 0.1% glutaraldehyde in PBS. After washing with PBS containing 50 mM glycine, cells were embedding in 12% gelatin and infused in 2.3 M sucrose. Mounted gelatin blocks were frozen in liquid nitrogen. Thin sections were prepared in an ultracryomicrotome (Leica EM Ultracut UC6/FC6, Vienna, Austria). Ultrathin cryosections were collected with 2% methylcellulose in 2.3 M sucrose. Cryosections were incubated at room temperature on drops of 2% gelatine in PBS for 20 min at 37°C, followed by 50 mM glycine in PBS during 15 min and 10% FBS in PBS during 10 min and 5% FBS in PBS 5 min. Then they were incubated with a mouse anti-ABCA1 and rabbit anti-caveolin 1 antibodies (1:50 both) in 5% FBS in PBS for 30 min. After three washes of PBS for 10 min, sections were incubated for 20 min with anti-mouse coupled to 12 nm and anti-rabbit coupled to 18 nm gold particles (Jackson ImmunoResearch, PA, USA). This was followed by three washes with drops of PBS for 10 min, two washes with distilled water. As a control for non-specific binding of the colloidal gold-conjugated antibody, the primary polyclonal antibody was omitted. The observations were done in an Electron Microscope Tecnai Spirit (FEI Company, The Netherlands) with a CCD camera SIS Megaview III.

### Cholesterol Efflux Assays

Peritoneal macrophages from WT and Cav-1^-/-^ mice were cultured at a density of 1 × 10^6^ cells per well 1 day prior to loading with 0.5 mCi/ml [^3^H]-cholesterol for 24 h with or without T0901317 (2 uM) for 12 h (35). Cells then were washed twice with PBS and incubated in RPMI 1640 medium supplemented with 2 mg/ml fatty acid-free bovine serum albumin (FAFA media) in the presence of an Acetyl-Coenzyme A Acetyltransferase (ACAT) inhibitor (2 mM; Novartis Corporation, New York, NY, USA) for 4 h prior to the addition of 50 ug/ml human ApoA1 in FAFA or HDL (Intracell) media. Supernatants were collected after 6 h and expressed as a percentage of [^3^H]-cholesterol in the media per total cell [^3^H]-cholesterol content (total effluxed [^3^H]-cholesterol + cell-associated [^3^H]-cholesterol).

## Results

### Caveolin-1 Expression Modulates Inflammatory Responses and ABCA1 Expression in RAW264.7 Cells

**Figure 1:**
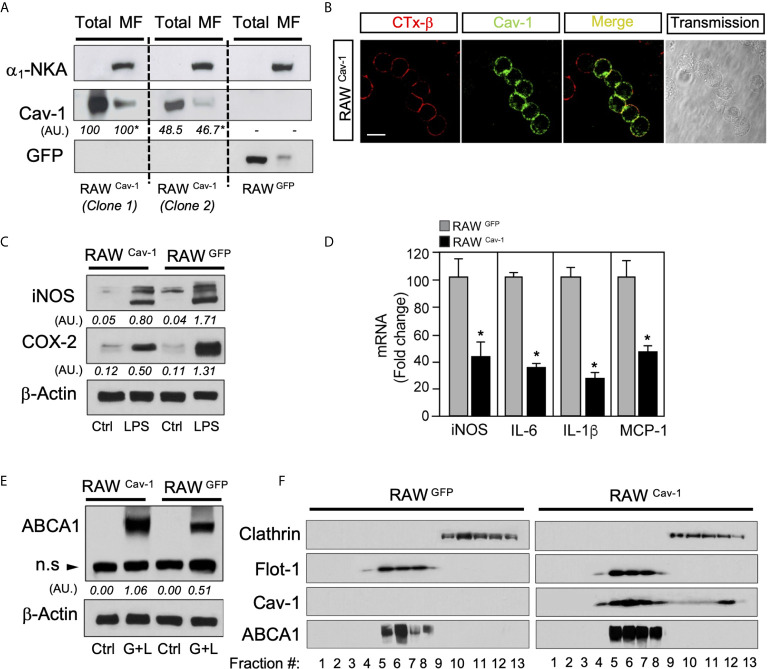
Caveolin-1 expression modulates inflammatory genes and ABCA1 expression in RAW264.7 cells. **(A)** Western blot analysis of ectopically expressed Cav-1 in RAW264.7 cells (RAW^Cav-1^) compared with RAW264.7 control cells overexpressing GFP (RAW^GFP^). α1-Na/K-ATPase protein was used to detect plasma membrane enrichment in microsomal preparations. Densitometric analysis of Cav-1 are shown below each blot and are referred to Total or MF in RAW^Cav-1^ cells clone 1. **(B)** Colocalization of Cav-1 with lipid rafts in the plasma membrane stained with CTx-β in RAW^Cav-1^cells. Scalebar: 10µm. **(C)** Representative Western blot analysis of iNOS and COX-2 in RAW^Cav-1^ and RAW^GFP^ cells. β-Actin was used as a loading control. Densitometric values of iNOS and COX-2 are shown below each blot. **(D)** qRT-PCR analysis of iNOS, IL-6, IL-1β and MCP-1 mRNA expression in RAW^Cav-1^ and RAW^GFP^ cells treated with 100ng/mL of LPS for 6 hours. Data are expressed as relative expression levels and correspond to the means ± SEM from three independent experiments performed in triplicate *P < 0.05 (significantly different from RAW^GFP^ cells). **(E)** Representative Western blot analysis of ABCA1 in RAW^Cav-1^ and RAW^GFP^ cells in response to LXR/RXR ligands for 24h(GW3965 1µM+LG268 100nM). β-Actin was used as a loading control. Densitometric values of ABCA1 are shown below each blot. **(F)** Representative Western blot analysis of lipid raft fractionation in RAW^Cav-1^ (left panel) and RAW^GFP^ (right panel) cells showing the expression of Cav-1 and ABCA1 in response to LXR/RXR ligands for 24h (GW3965 1µM+LG268 100nM). Flotillin-1 was used as positive control of raft fractions and Clathrin as non-raft protein.

Previous studies have shown that ligand-activated LXRs down-regulate the expression of inflammatory genes in macrophages (6, 36–38), and other studies identified Cav-1 as an immunomodulatory mediator in these cells (39, 40). In order to explore the possible implication of Cav-1 in the modulation LXR anti-inflammatory function, we analyzed the LPS-induced cytokine production in various clones of RAW264.7 macrophages expressing Cav-1 ectopically. RAW264.7 control cells (expressing green fluorescent protein, GFP) do not express Cav-1 basally (**Figure 1A**). Based on the expression levels of Cav-1 in purified fractions of plasma membrane assessed by Western blot (**Figure 1A**), and the co-localization with GM-1 ganglioside, a marker of lipid rafts labelled with a fluorescent choleric β-subunit toxin, we chose RAW Cav-1 clone 1 for further analysis (**Figure 1B**). Western blot and quantitative real-time PCR (qRT-PCR) experiments showed that LPS-dependent expression of iNOS and COX-2 (**Figure 1C**), as well as IL-6, IL-1β and MCP-1 (**Figure 1D**) was reduced in Cav-1 overexpressing cells compared with control GFP cells. Next, we explored whether Cav-1 expression could influence the expression of LXR target genes. RNA expression of Abca1, Abcg1, Apoe and Srebf1 was similar in RAW-GFP and RAW-Cav-1 cells (not shown). Since CAV-1 function has been associated to cholesterol trafficking within the plasma membrane, we explored whether expression of Cav-1 in RAW cells influences the localization of the key protein involved in cholesterol efflux ABCA1. Interestingly, we observed higher levels of ABCA1 protein in whole cell lysates of Cav-1 overexpressing macrophages compared to control cells (**Figure 1E**), as well as in lipid raft fractionation experiments, were ABCA1 co-fractionated with Cav-1 and other markers like Flotillin-1 (Flot-1) in rafts membranes (**Figure 1F**). Together, these results indicate that ectopic expression of Cav-1 in RAW264.7 cells reduced inflammatory gene expression and promotes ABCA1 protein localization within membrane raft microdomains. Altogether these results indicate that LXR and Cav-1 functions exhibit a reciprocal influence on each other and suggest a possible cross-talk between LXR and Cav-1 signaling in macrophages.

### Caveolin-1 Expression Is Reduced in LXRαβ^-/-^ Mouse Peritoneal Macrophages and Liver

**Figure 2:**
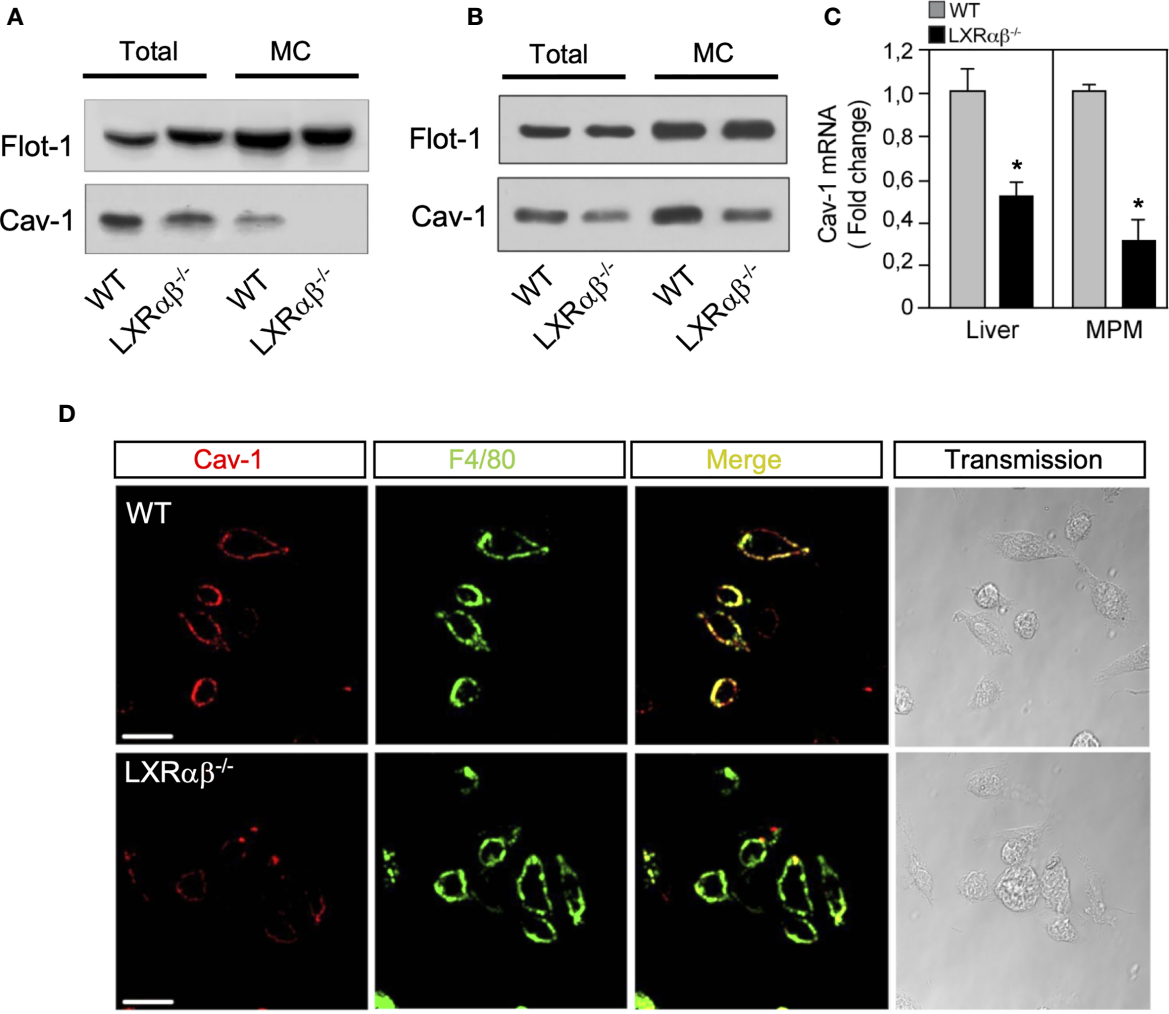
Cav-1 expression is impaired in LXR*αβ^-/-^* mice. Representative Western blot analysis of Cav-1 in whole-cell lysates (Total) and microsomal fractions (MC) of peritoneal macrophages **(A)** and liver **(B)** from in WT and *LXRαβ^-/-^* mice. **(C)** mRNA expression of Cav-1 by real-time qRT-PCR in peritoneal macrophages and liver from WT and *LXRαβ^-/-^* mice. Data are expressed as relative expression levels and correspond to the means ± SEM from three independent experiments performed in triplicate *P < 0.05 (significantly different from WT [normalized to 1]). **(D)** Representative confocal images of Cav-1 expression (red) and F4/80 (green) in peritoneal macrophages from WT and *LXRαβ^-/-^* mice. Experiment was performed 3 independent times. Scalebar: 10µm.

Since Cav-1 overexpression enhanced ABCA1 protein in lipid rafts and promoted anti-inflammatory effects in macrophages, we assessed the abundance and distribution of Cav-1 in WT and *LXRαβ^-/-^* deficient mice. Our Western blot analysis in total cell lysates and in microsomal extracts from mouse peritoneal macrophages showed reduced Cav-1 expression in *LXRαβ^-/-^* cells compared to WT (**Figure 2A**), whereas no changes were observed in other lipid rafts marker such as Flot-1. Additionally, Cav-1 expression was also decreased in liver extracts from in *LXRαβ^-/-^* compared to WT mice (**Figure 2B**). Further, real-time qPCR analysis revealed a reduction of approximately 50% of Cav-1 mRNA levels in both peritoneal macrophages and liver samples from *LXRαβ* deficient mice (**Figure 2C**). Then, to better characterize the localization of Cav-1 within the plasma membrane, we observed the expression of the macrophage-specific membrane antigen F4/80 and Cav-1 in WT and *LXRαβ^-/-^* macrophages. Confocal images showed double positive immunostaining of F4/80 and Cav-1 in WT peritoneal macrophages, while the colocalization of both proteins was less evident in *LXRαβ^-/-^* macrophages due to reduced expression of Cav-1 in these cells (**Figure 2D**). Similar results were found in Kupffer cells from liver sections of *LXRαβ^-/-^* mice compared to WT controls (**Supplementary Figure 1**). These results indicate that LXR activity is important for the plasma membrane localization of Cav-1 in macrophages.

### Subcellular Distribution Caveolin-1 and ABCA1 Is Controlled by LXR activity

**Figure 3:**
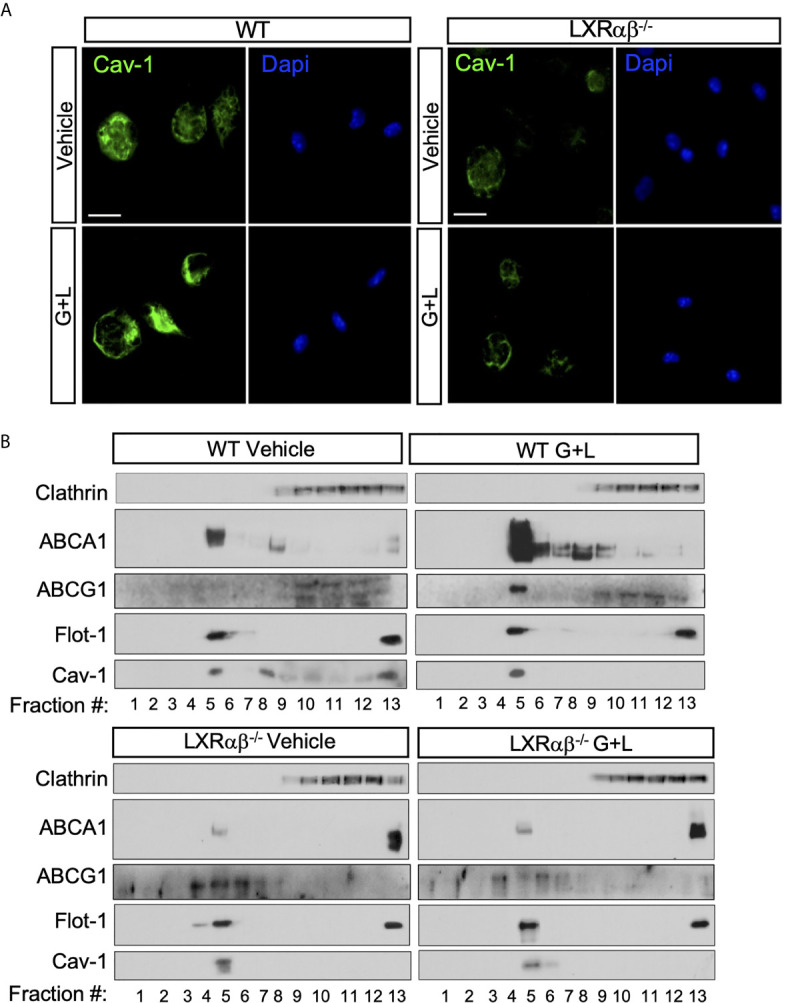
Cav-1 cellular distribution is dependent on LXR. **(A)** Representative images showing the subcellular distribution of Cav-1 (green) by confocal microscopy in peritoneal macrophages from WT and *LXRαβ^-/-^* mice treated for 24 h with 1 μM GW3965 and 100 nM LG268 (G+L). Nuclei were stained with DAPI. Experiment was performed 3 independent times. Scalebar: 10µm. **(B)** Representative Western blot analysis of Cav-1 and ABCA1 and ABCG1 in lipid raft fractions from WT and *LXRαβ^-/-^* peritoneal macrophages treated for 24 h with G+L.

Previous studies have reported contrasting data regarding the regulation of Cav-1 functions by cellular cholesterol in different mouse or human macrophage cell models (41). However, given the well-known role for Cav-1 in cholesterol transport from endoplasmic reticulum to the plasma membrane and the recent identification of the LXR targets, Gramd1/Aster, that control the movement of cholesterol accessible pools within the plasma membrane (42–44), we decided to explore whether LXR activation would affect Cav-1 expression and subcellular distribution in macrophages. To this end, we first performed microscopy analysis to examine the patterns of Cav-1 immunoreactivity within the plasma membrane during LXR activation. Interestingly, cells treated with a combination of the synthetic LXR and RXR ligands (GW3965 and LG268; GW+LG) showed an intense staining of plasma membrane Cav-1 compared to the vehicle-treated cells (**Figure 3A**, left panel), while such increase was not observed in LXR-null macrophages (**Figure 3A**, right panel). These results suggested a possible subcellular relocalization of Cav-1 in response to LXR activation. To further characterize the changes in Cav-1 localization in response to LXR activation, we used a sodium carbonate detergent-free method to purify lipid rafts from peritoneal macrophages. Western blot analysis from sucrose gradient fractions using specific antibodies against Cav-1, Flot-1, ABCA1 and ABCG1 showed that Cav-1 protein expression appeared mainly at light buoyant fraction (F4-F5) containing the lipid raft membranes together with the raft marker Flot-1, as well as in other intracellular organelles and membranes in peritoneal macrophages (**Figure 3B**, upper panel). Interestingly, after LXR activation, Cav-1 expression was slightly increased in the raft fraction but decreased in the non-raft compartments, consistent with an intracellular redistribution of Cav-1 dependent on LXR. Importantly, these effects of Cav-1 redistribution within raft fractions were abolished in *LXRαβ^-/-^* macrophages (**Figure 3B**, lower panel). Next, we also assessed the expression and localization of ABCA1 and ABCG1 under the same sucrose fractioning conditions. Western blot analysis showed that ABCA1 and ABCG1 proteins were mostly present in the lipid raft fraction and it co-fractionated with Cav-1 in wild type macrophages but not in *LXRαβ^-/-^* macrophages (**Figure 3B**, both top and lower panels). Moreover, in agreement with role of Cav-1 as a cholesterol transport protein and the capacity of LXR to control cholesterol pools within the cell, LXR activation promoted subcellular redistribution of cholesterol (stained with Filipin III), together with Cav-1, in WT macrophages but it remained and accumulated intracellularly in *LXRαβ^-/-^* cells (**Supplementary Figure 2**). This may represent an abnormal localization of this protein within the macrophage in cells lacking both isoforms of LXR. Together, these results indicate that LXR activation promotes ABCA1, ABCG1 and Cav-1 relocalization within the raft microdomains of the plasma membrane in murine macrophages.

### Cav-1 and ABCA1 Co-Localize in Mouse Peritoneal Macrophages

**Figure 4:**
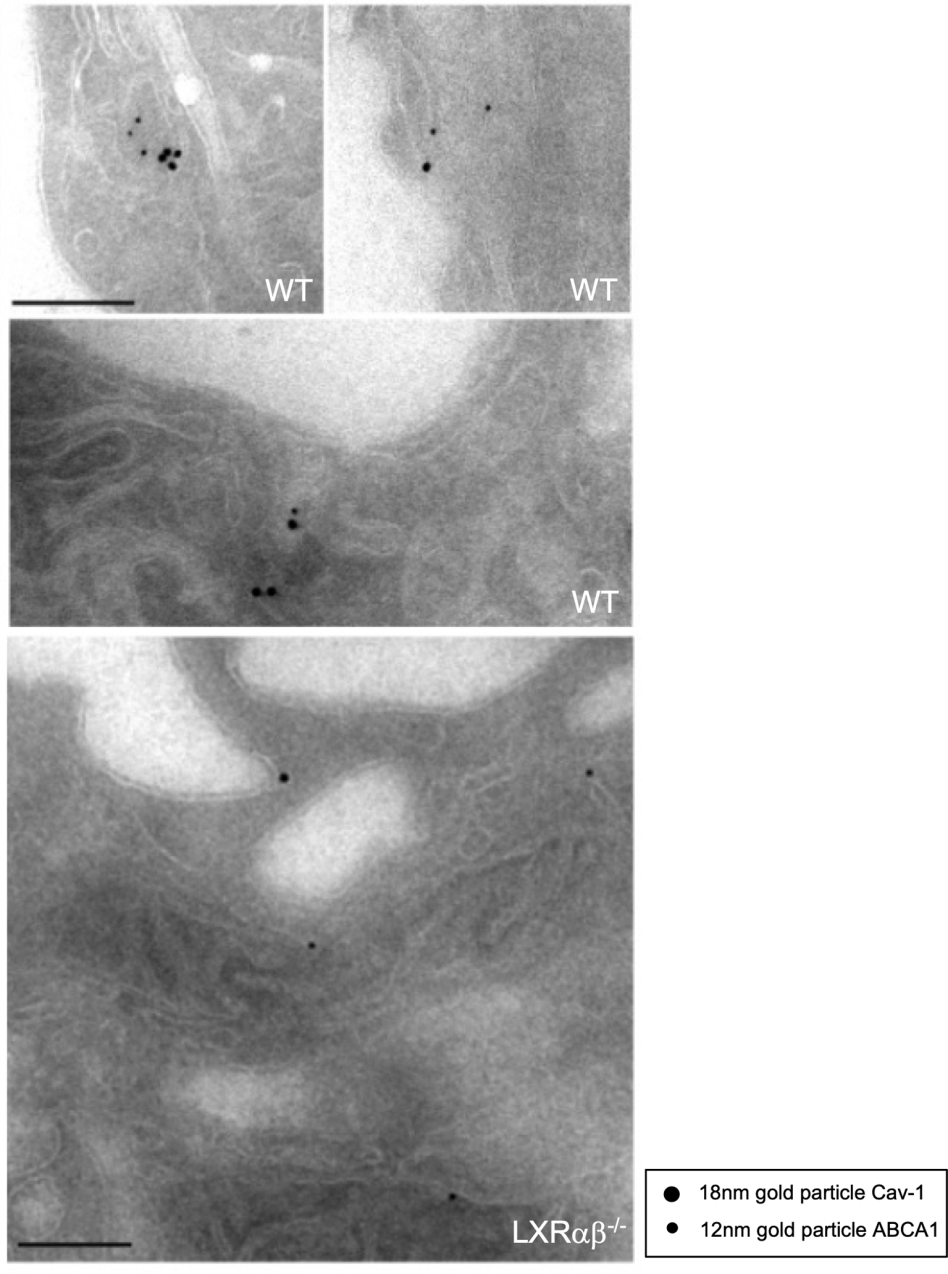
Colocalization of Cav-1 and ABCA1 within sub-cellular domains of peritoneal macrophages. Representative immuno-electron microscopy images showing Cav-1 and ABCA1 expression in peritoneal macrophages from WT and *LXRαβ^-/-^* mice. Scalebar: 200nm.

Despite of the well-established importance of ABCA1 in cholesterol transport and role of Cav-1 in the cholesterol homeostasis, the possible colocalization of these two proteins has not been fully identified at the ultrastructural level in macrophages. Based on our previous results showing the co-fractionation of ABCA1 with Cav-1 we decided to further characterize in depth the colocalization of both proteins in mouse macrophages. To do so, we performed electron microscopy studies of colloidal ABCA1 and Cav-1 gold particles. Individual immunostainings revealed that both particles were primarily located in the caveolae-like structures and intracellular cytoplasmic vesicles, as well as in other non-caveolae membranes (**Supplementary Figure 3**). We then performed combined labeling of ABCA1 and Cav-1 in WT and *LXRαβ^-/-^* macrophages. **Figure 4** (upper micrographs) shows that ABCA1 and Cav-1 were consistently found decorating the membrane of the same vesicles. Intriguingly, we were not able to find colocalization of immunogold particles of ABCA1 and Cav-1 in *LXRαβ^-/-^* macrophages **Figure 4** (lower micrographs). These results are in agreement with our previous co-fractionation of Cav-1 and ABCA1 and suggest a possible reciprocal relationship of their functionalities, with possible implications of Cav-1 in LXR-dependent regulation of cholesterol metabolism and inflammation.

### The Anti-Inflammatory Actions of LXR Agonists Are Decreased in Cav-1^-/-^ Macrophages

**Figure 5:**
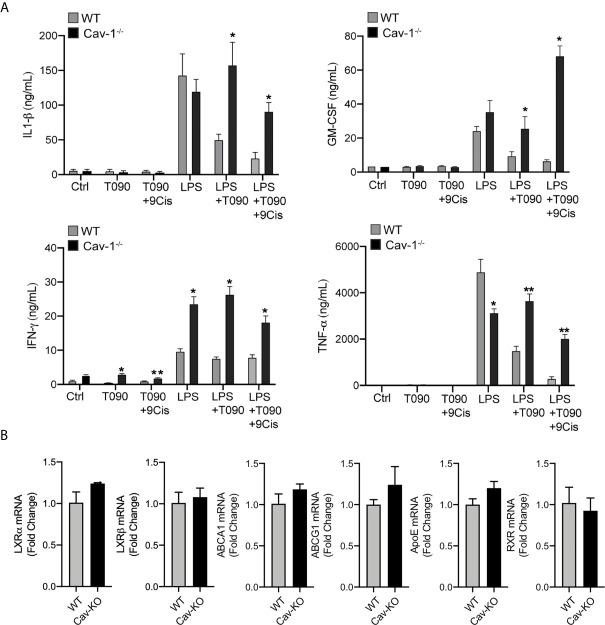
Cav-1 influences anti-inflammatory effects of LXR in peritoneal macrophages. **(A)** Cytokine production in culture media of peritoneal macrophages from WT and *Cav-1^-/-^* mice pre-treated with 1 μM T0901317 and 1 μM 9-Cis Retinoic Acid for 18h hours prior to stimulation with LPS for another 24h. Data represent 3 independent experiments performed in triplicate *P < 0.05, **P < 0.01 (significantly different from WT in Ctrl conditions). **(B)** qRT-PCR analysis of LXRα, LXRβ, ABCA1, ABCG1 and ApoE mRNA levels in peritoneal macrophages form WT and *Cav-1^-/-^* mice. Data are expressed as relative expression levels and correspond to the means ± SEM from three independent experiments performed in triplicate compared to WT and normalized to 1).

We and others have previously reported that cultured WT macrophages with LXR agonists display reduced expression of inflammatory markers in response to LPS. These anti-inflammatory actions are abolished in macrophages from *LXRαβ^-/-^* mice (38, 45). In addition, given that overexpression of Cav-1 in macrophages partially inhibited inflammatory cytokine production and LXR activation promoted Cav-1 redistribution within the raft membrane domains, we hypothesized that Cav-1 could be involved in LXR-dependent regulation of inflammation. To asses this, we compared the inflammatory outcomes of primary peritoneal macrophages from wild type (WT) and *Cav-1^−/−^* mice treated with LPS alone, or in combination with LXR/RXR ligands. Interestingly, we found that the inhibitory capacity of LXR to block the production of pro-inflammatory cytokines, including TNF-α and IL-1β, as well as other mediators such as IFN−γ or GM-CSF was severely compromised in *Cav-1^-/-^* macrophages (**Figure 5A**). Similar results were observed by analyzing iNOS and COX-2 expression by Western blotting (not shown). Nevertheless, the ameliorated anti-inflammatory response of LXR in the absence of Cav-1 was not due to differences in the expression of LXRα, LXRβ or their target genes in Cav-1 deficient cells (**Figure 5B**). These results suggest that Cav-1 expression is an important element that facilitates full acquisition of LXR anti-inflammatory signaling in macrophages.

### Cholesterol Efflux to ApoAI and HDL Is Impaired in Cav-1 Deficient Macrophages

**Figure 6:**
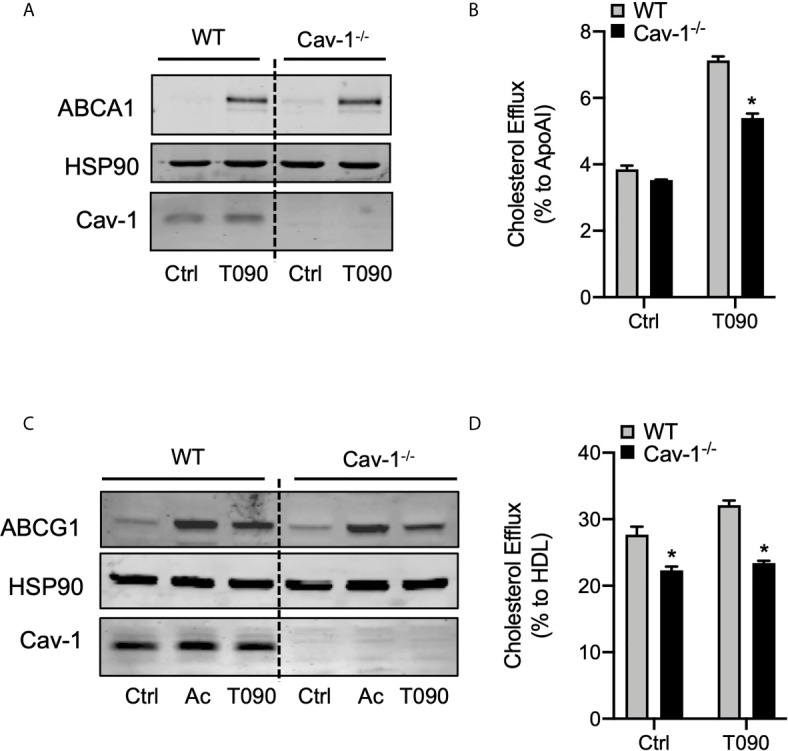
Cav-1 influences subcellular distribution of ABCA1 and modulates ABCA1-dependent cholesterol efflux. **(A)** Western blot analysis of ABCA1 in peritoneal macrophages from WT and *Cav-1^-/-^* mice treated with or without or 1 μM T0901317 (T090) for 18 hours. Cav-1 was used to show its absence in the *Cav-1^-/-^* mice and HSP90 was used as a loading control. Experiment was performed at least 3 times. **(B)** Cholesterol efflux to apolipoprotein A1 (ApoA1) in peritoneal macrophages isolated from WT and *Cav-1^-/-^* mice stimulated with or without 3 μM of T090 for 16 hours. Data represent the mean ± SEM of triplicate samples (n = 3 per group; *P < 0.05*, (significantly different from WT in each treatment condition). **(C)** Western blot analysis of ABCG1 in peritoneal macrophages from WT and *Cav-1^-/-^* mice treated with or without or 1 μM T0901317 (T090) for 18 hours. Cav-1 was used to show its absence in the *Cav-1^-/-^* mice and HSP90 was used as a loading control. Experiment was performed at least 3 times. **(D)** Cholesterol efflux to HDL in peritoneal macrophages isolated from WT and *Cav-1^-/-^* mice stimulated with or without 3 μM of T090 for 16 hours. Data represent the mean ± SEM of triplicate samples (n = 3 per group; *P < 0.05*, (significantly different from WT in each treatment condition). *P < 0.05.

The induction of ABCA1 and ABCG1 expression and activation of the cholesterol efflux pathway (2, 3), together with inhibition of inflammation, are considered crucial steps for the atheroprotective functions of LXRs. Because our results suggest that ABCA1 and Cav-1 proteins co-localize in cellular membranes and that this mutual cooperation is promoted by LXR activity, we decided to explore whether alteration of Cav-1 expression could influence cholesterol efflux in macrophages. First, we assessed the expression of ABCA1 and ABCG1 in WT and Cav-1-/- macrophages. Although ABCG1 levels were slightly different in Cav-1 null macrophages, induction of both ABC transporters was appreciable in response to LXR agonists in WT and Cav-1 KO macrophages (**Figures 6A, C**). Next, we assayed *in vitro* the cholesterol efflux to ApoAI and HDL in primary peritoneal macrophages from WT and Cav-1-deficient mice. As shown in **Figures 6B, D**, the cholesterol efflux to ApoAI and HDL was significantly blunted in *Cav-1^-/-^* macrophages, indicating that Cav-1 expression is important for LXR-dependent cholesterol efflux. Similar results were obtained in bone marrow derived macrophages (not shown). Our results show that activation of LXR induced the co-expression of ABCA1, ABCG1 and Cav-1 in lipid raft microdomains within the plasma membrane. Since the overall ABCA1 protein content was similar to WT in Cav-1^-/-^ macrophages, we reasoned whether lack of Cav-1 might have an influence in ABCA1 distribution within the membrane. To analyze this possibility, we cultured macrophages with LXR/RXR ligands and observed the localization of ABCA1 by confocal microscopy. As shown in **Supplementary Figure 4**, LXR activation induced a punctuate accumulation of ABCA1 protein in the membrane of WT macrophages, whereas this distribution was severely altered in Cav-1^-/-^ macrophages. Taken together, this data indicate that Cav-1 participates in the distribution of ABCA1 within the plasma membrane and in ApoAI-dependent cholesterol efflux in response to LXR activation in macrophages.

## Discussion

LXRs are transcription factors that regulate crucial processes in lipid metabolism and also exert important functions in inflammation and host immunity (46, 47). LXRs have emerged as key regulators of whole body cholesterol homeostasis, in part through up-regulation of genes encoding plasma membrane transporters ABCA1 and ABCG1 that facilitate cholesterol efflux from macrophages and other cell types (1, 2, 48). Importantly, the same pathways that control cholesterol trafficking within the plasma membrane appeared as important regulators of inflammation and host defense (49, 50). In line with this, regulation of cholesterol efflux and the control of inflammatory and antimicrobial responses in macrophages have been shown to be partially dependent on ABCA1 (7, 50, 51). In the present study we provide an additional mechanistic clue that identifies Cav-1 as an important factor required for LXR-dependent functions in murine macrophages. Both the anti-inflammatory actions and cholesterol efflux to ApoAI or HDL promoted by LXR agonists are substantially diminished in the absence of Cav-1. Reciprocally, localization of Cav-1 within lipid-raft membrane microdomains is impaired in LXR-deficient macrophages. These findings reveal that a mutual cooperation between Cav-1 and LXR pathways participates in macrophage inflammatory and homeostatic responses.

Among caveolin proteins, Cav-1 is crucial for the formation of caveolae and participates in several cellular processes, including endocytosis, cholesterol trafficking and signal transduction (52, 53). However, the role of Cav-1 during atherosclerosis is context and cell-type dependent. Hypercholesterolemic mice with complete loss of Cav-1 or with Cav-1 targeting specifically in endothelial cells demonstrated that Cav-1 function accelerates atherogenesis by modulating endothelial lipoprotein transport and autophagy pathways (54, 55). In contrast, other studies using bone marrow transplant of *Cav-1* ^-/-^ hematopoietic progenitors into *LDLR* ^-/-^ mice indicated that an important contribution of Cav-1 in macrophages is generally atheroprotective through modulation of macrophage inflammation (56). Thus, these reports reconcile the apparent contrasting roles of Cav-1 in atherosclerosis and highlight the complexity and the importance of Cav-1 in lipid and inflammatory pathways in the context of atherosclerosis. Also, all these studies infer that LXRs and Cav-1 appear to participate in similar pathways in macrophages, including phagocytosis, inflammation and cholesterol efflux. However, a deeper comprehension of how LXR and Cav-1 pathways can affect each signaling reciprocally was not explored before.

The first experimental evidence that supported a mutual crosstalk between Cav-1 and LXR came from our experiments of forced Cav-1 expression in RAW264.7 macrophages that normally lack Cav-1. In this model, Cav-1 favors accumulation of ABCA1 mainly in lipid-raft domains in response to LXR ligands and a marked attenuation of inflammatory gene expression in response to LPS (**Figure 1**). Conversely, Cav-1 expression exhibited a reduction of ~50% in mRNA and total and microsomal protein expression in LXR-deficient macrophages and liver, compared WT controls (**Figure 2**). Cav-1 down-regulation in LXR-null macrophages possibly suggests that LXR might be controlling Cav-1 expression through transcriptional regulation. However, our previously reported LXR ChIP-Seq and RNA profiling studies (57), have not demonstrated direct LXR binding in the vicinity of the Cav-1 locus nor direct up-regulation of Cav-1 expression in response to LXR ligands, indicating that impaired Cav-1 expression in LXR-null macrophages could be the result of an indirect regulation. Our observations indicate that ABCA1 and ABCG1 are concomitantly recruited with Cav-1 to lipid-raft microdomains (**Figure 3**). Our results are consistent with previous studies that showed a role for Cav-1 in ABCA1 and ABCG1 function in other cell types (58, 59). Thus, the occurrence of Cav-1 in lipid-rafts of the plasma membrane augmented in response to LXR activation in macrophages and we hypothesize that the critical cholesterol efflux transporters ABCA1 and ABCG1 are important for this regulation. However, it is also possible that the combined induction of several other direct LXR-regulated targets in response to elevated cholesterol levels assist in Cav-1 recruitment to the plasma membrane in macrophages. Recently, elegant studies from Tontonoz and colleagues identified a family of proteins, called Aster, that facilitate the transport of excess accessible cholesterol in the plasma membrane to the endoplasmic reticulum in response to LXR activation (42, 44). Thus, although the factors that are responsible for Cav-1 down-regulation in LXR-deficient macrophages need further investigation, our observations indicate that an intact LXR signaling is crucial for Cav-1 subcellular localization within the plasma membrane in macrophages.

Previous reports have described that overexpression of ABCA1, ABCG1 and ABCG4 in HEK293 and CHO cells influences cholesterol efflux through their activity in non-raft domains (60), based on experiments using detergents that solubilize lipid membrane domains. Our experiments in primary macrophages, however, show that the majority of ABCA1 co-fractioned mainly with Cav-1 in light buoyant or caveolar fractions. Furthermore, we demonstrate that ABCA1 and ABCG1 induction promoted by LXR activation takes place mainly on caveolar fractions along with other lipid-rafts markers such as flotillin-1 (61). Our ultrastructural analyses (**Figure 4**) also indicate that ABCA1 and Cav-1 do not appear to interact physically but consistently co-localize within common membrane microdomains. Importantly, our observations demonstrate that loss of LXR in macrophages, that greatly increases the intracellular cholesterol content (62), leads to reduced Cav-1 and ABCA1 recruitment to lipid-rafts possibly by disrupting caveolae microdomains. Our conclusions are in agreement with previous results by Parks and colleagues that showed increased membrane lipid raft content with reduced Cav-1 in raft fractions and hyper-inflammatory responses in ABCA1-deficient macrophages (63). Our studies demonstrate that LXR deficiency in macrophages directs a reduction of Cav-1 within the lipid-raft membrane domains that would promote delocalization of ABCA1 from raft/caveolar to non-raft membranes and other subcellular locations. These molecular changes would probably influence the normal ABCA1 functions in macrophages. ABCA1 activity is critical for cholesterol efflux and was also found to play anti-inflammatory tasks in macrophages (7, 50, 63). Our data indicate that some of the LXR-dependent functions that are critically mediated by ABCA1, appear to rely on Cav-1 expression. Using Cav-1 deficient macrophages, we demonstrate that the ability of LXR ligands to control the expression of inflammatory mediators depends greatly on Cav-1 expression. These results are congruent with the studies by Ito et al. (7), which clearly showed that induction of ABCA1 and subsequent uncoupling of TLR signaling from the plasma membrane mediate the anti-inflammatory actions of LXR. It seems plausible from these studies and our results that Cav-1 and ABCA1 participate in the regulation of inflammation by controlling the recruitment of adaptor molecules that mediate inflammation downstream of TLR. When the connection between ABCA1 and Cav-1 within lipid-rich microdomains is enhanced by pre-treatment with LXR ligands, the magnitude of inflammatory responses is attenuated. Furthermore, the importance of Cav-1 expression in LXR-ABCA1 functions in macrophages has also implications in cholesterol efflux. Cav-1 deficiency results in a marked reduction in LXR-dependent cholesterol efflux to ApoA-I and HDL in macrophages. The defect in cholesterol efflux is not due to defect in ABCA1 or ABCG1 total protein expression, nor defects in LXRα or LXRβ expression in Cav-1^-/-^ macrophages. It is likely that the disruption of caveolar microdomains by the loss of Cav-1 (64) affects the membrane localization and function of ABCA1 and perhaps other proteins regulated by LXR involved in cholesterol efflux. Overall, our studies conclude that Cav-1 plays an important role in LXR-mediated functions, both in inflammation and in cholesterol efflux and that Cav-1 functions in macrophages could provide additional intervention mechanisms to the LXR transcriptional regulation of cholesterol efflux and inflammation.

## Data Availability Statement

The raw data supporting the conclusions of this article will be made available by the authors, without undue reservation.

## Ethics Statement

The animal study was reviewed and approved by Comité Etica y experimentación animal Consejo Superior de Investigaciones Científicas and Universidad Las Palmas de Gran Canaria, with reference numbers PROEX171/2018 and OEBA-ULPGC 02/2015.

## Author Contributions

CMR and AC conceived the project. CMR, LB and AC contributed to the conceptual design, figure preparation and acquired the funding for the project. CMR, MTP, VPM, MFF, APG, CT, JVR, PMR, MDS, UN, MCO, PGT and MC performed experiments and data analysis. CMR and AC prepared draft manuscript. All authors revised the draft manuscript and contributed to its editing. All authors contributed to the article and approved the submitted version.

## Conflict of Interest

The authors declare that the research was conducted in the absence of any commercial or financial relationships that could be construed as a potential conflict of interest.

The handling editor declared a past co-authorship with one of the authors AC.

## Supplementary Material

Supplementary Figure 1 LXR deficiency in the liver results in decreased Cav-1 expression in Kupffer cells. Representative confocal images of Cav-1 expression (red) and F4/80 (green) in livers from WT and *LXRαβ^-/-^* mice. Higher magnification of the insets is shown in the right panels. Scalebar: 20µm.

Supplementary Figure 2 Cellular cholesterol redistribution upon LXR activation correlates with Cav-1 localization in peritoneal macrophages. Representative images showing the subcellular distribution of Cav-1 (green) and Fillipin (blue) in peritoneal macrophages isolated from WT and *LXRαβ^-/-^* mice and treated for 24 h with 1 μM GW3965 and 100 nM LG268 (G+L). Scalebar: 10µm.

Supplementary Figure 3 Cav-1 and ABCA1 localization in caveolae-like and intracellular vesicles. Representative electron microscopy images showing individual immunogold staining of Cav-1 and ABCA1 in peritoneal macrophages isolated from WT and *LXRαβ^-/-^* mice. Scalebar: 200nm.

Supplementary Figure 4 Cav-1 influences subcellular distribution of ABCA1 Representative images showing immunostaining of ABCA1 (red) by confocal microscopy in peritoneal macrophages from WT and *Cav-1^-/-^* mice treated for 24 h with 1 μM GW3965 and 100 nM LG268 (G+L). Nuclei were stained with DAPI. Experiment was performed 3 independent times. Scalebar: 20µm.
