# Supplementary figures and images for: Crosstalk Between LXR and Caveolin-1 Signaling Supports Cholesterol Efflux and Anti-Inflammatory Pathways in Macrophages
Source: Front Endocrinol (Lausanne). 2021 May 27;12:635923. doi: 10.3389/fendo.2021.635923 (PMC8190384; doi:10.3389/fendo.2021.635923)

# Supplementary Figure 1

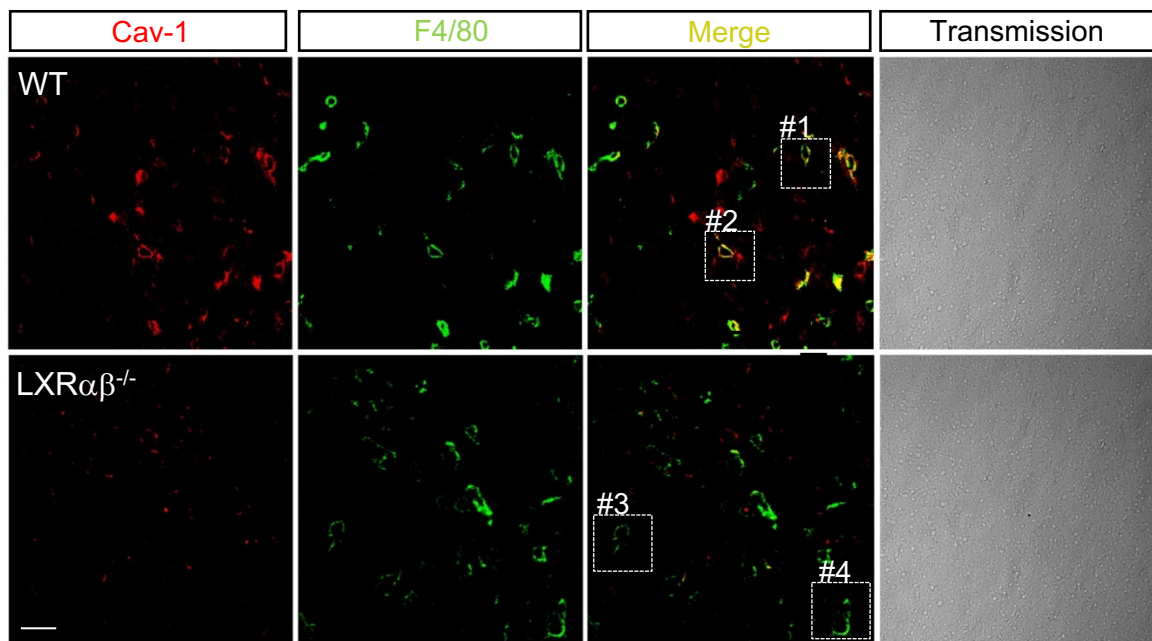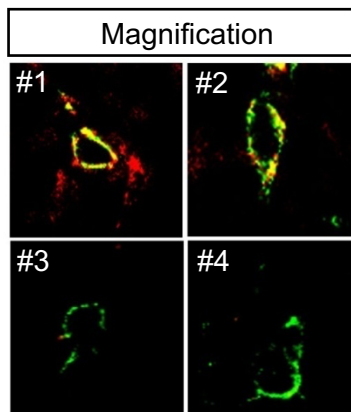

Supplement: Supplementary Figure 1 — LXR deficiency in the liver results in decreased Cav-1 expression in Kupffer cells. Representative confocal images of Cav-1 expression (red) and F4/80 (green) in livers from WT and LXRαβ-/- mice. Higher magnification of the insets is shown in the right panels. Scalebar: 20µm. [file DataSheet_1.pdf]

# Supplementary Figure 2

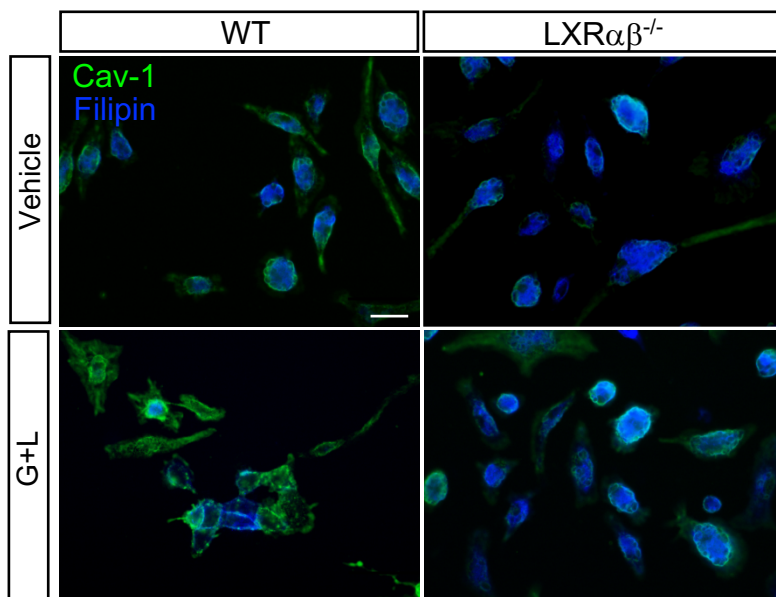

Supplement: Supplementary Figure 2 — Cellular cholesterol redistribution upon LXR activation correlates with Cav-1 localization in peritoneal macrophages. Representative images showing the subcellular distribution of Cav-1 (green) and Fillipin (blue) in peritoneal macrophages isolated from WT and LXRαβ-/- mice and treated for 24 h with 1 μM GW3965 and 100 nM LG268 (G+L). Scalebar: 10µm. [file DataSheet_2.pdf]

# Supplementary Figure 3

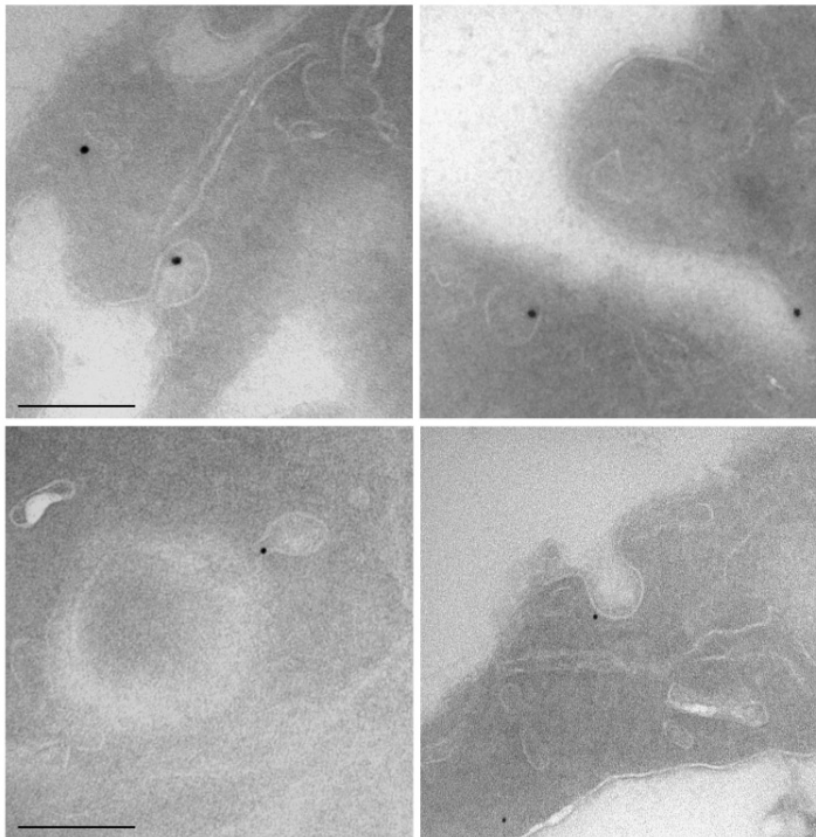

- 18nm gold particle Cav-1
- 12nm gold particle ABCA1

Supplement: Supplementary Figure 3 — Cav-1 and ABCA1 localization in caveolae-like and intracellular vesicles. Representative electron microscopy images showing individual immunogold staining of Cav-1 and ABCA1 in peritoneal macrophages isolated from WT and LXRαβ-/- mice. Scalebar: 200nm. [file DataSheet_3.pdf]

Supplementary Figure 4

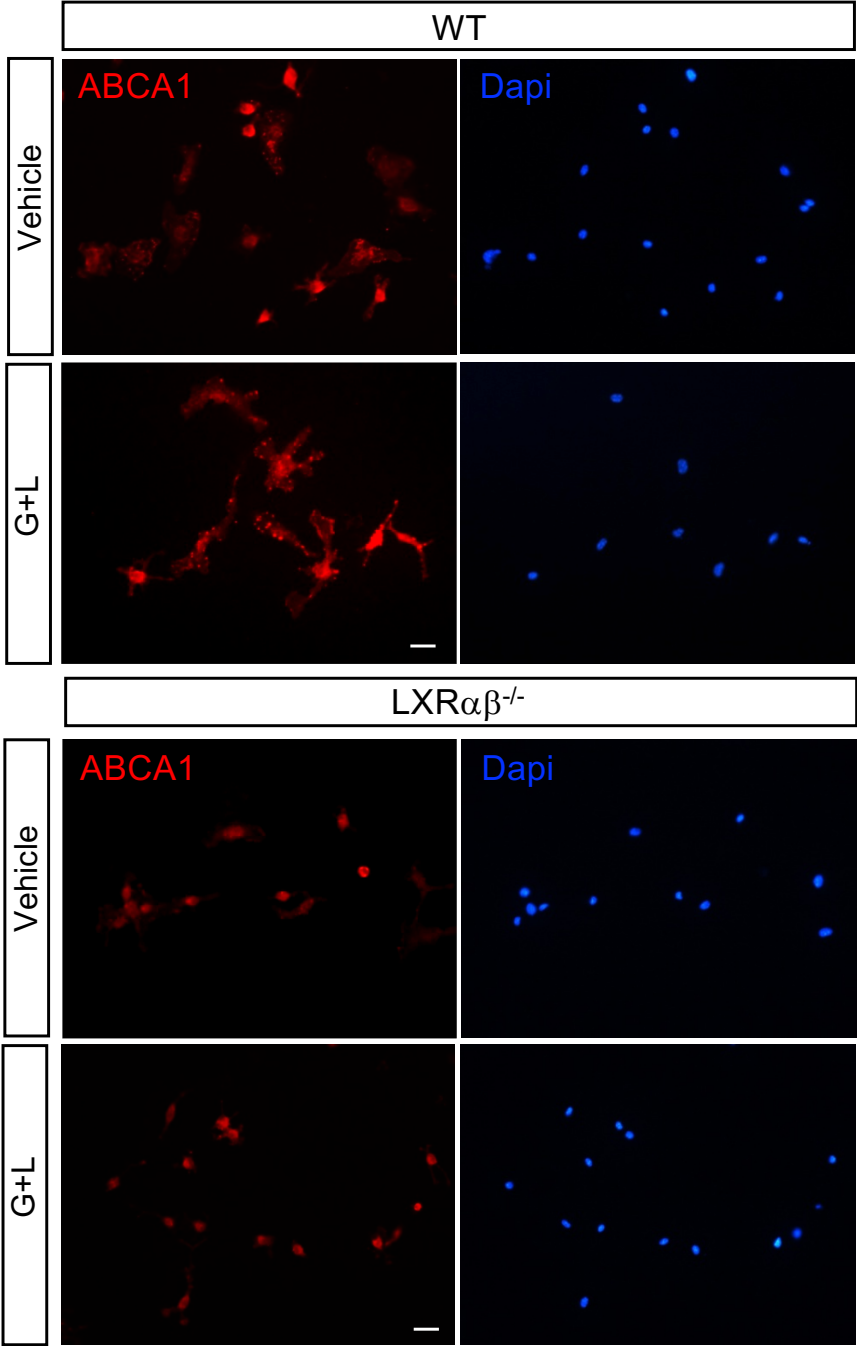

Supplement: Supplementary Figure 4 — Cav-1 influences subcellular distribution of ABCA1 Representative images showing immunostaining of ABCA1 (red) by confocal microscopy in peritoneal macrophages from WT and Cav-1-/- mice treated for 24 h with 1 μM GW3965 and 100 nM LG268 (G+L). Nuclei were stained with DAPI. Experiment was performed 3 independent times. Scalebar: 20µm. [file DataSheet_4.pdf]
